# Navigating Professional and Personal Knowing Through Reflective Storytelling Amidst Covid-19

**DOI:** 10.1177/08980101211072289

**Published:** 2022-12

**Authors:** Margaret M. Graham

**Keywords:** reflection, storytelling, caring and compassion, nurse education, nurse educator

## Abstract

The paper offers space for dialogue illustrating reflection as lived, exploring both my personal and professional experiences of grief and loss surrounding the death of my Dad from Covid −19. In my role as a nurse educator, I share understandings of reflection in facilitating learning and person centered practices with students. I illustrate my approach with two stories generating a narrative giving testimony to those who have died and highlighting the ensuing grief for those who have cared for older people during the pandemic. The first reflective story has been shared with students and snapshots of student responses during virtual sessions are incorporated. The second story shifts to a more personal focus reflecting personal knowing. Insights emerge bringing forth personal and professional knowing, about the art and science of holistic nursing. I explore the challenges in separating ourselves from personal knowledge and experience in reflective writing. I invite readers to take time to pause amidst a global healthcare pandemic to consider the potential of reflection to support nurses in recovering from suffering experienced during a pandemic.

## Foreword

I begin by introducing my background and vision as a nurse educator which foregrounds stories drawing from my reflective journaling in the midst of the new chaos of our world. Times likened by the English novelist Dickens (1859) writing in *Tale of Two Cities* about life during the French revolution on human conflict, and kindness - a tale of opposites without any in-betweens:

‘It was the best of times, it was the worst of times, it was the age of wisdom, it was the age of foolishness, it was the epoch of belief, it was the epoch of incredulity, it was the season of light, it was the season of darkness, it was the spring of hope, it was the winter of despair…’. (p.4)

Words, mirroring my thoughts as a lecturer at a nursing department at an Irish University, in an epoch of a pandemic. Central to my teaching and scholarly activity is reflection as a learning approach aiming to explore how we relate to human beings. I aim to foster holistic nursing as a lifelong commitment to person centered nursing with students.

Person-centredness is a multifaceted approach. It is an approach that moves away from a disease and medically orientated emphasis to relationship focused, collaborative, and holistic nursing. It is underpinned by core values of respect, understanding, dignity and compassion. Dewing and McCormack (2017) acknowledge that refining such ethereal terms is an ever evolving practice development challenge. One way of clarifying and fostering individual and collective person centered practices is reflection. As Clarke (2014) reports, reflection is about subjective experience in getting to know oneself, a crucial process encouraging reflectors to be person-centered therapists.

Nurse educators, while acknowledging the potential of reflection, do not necessarily practice reflection (Grech, 2021). In addressing such concerns I gained a deeper understanding of reflection through constructing a self-study narrative inquiry as designed by Johns (2010). My work is influenced by diverse sources including auto-ethnography (Ellis & Bochner, 2004), critical social theory (Fay, 1987), hermeneutics (Gadamer, 1989), reflection (Schön, 1983) and literary texts. Insights gained through self-inquiry illuminate my vision in fostering learning spaces in enabling students to reach their potential in becoming skilled and holistic practitioners (Graham & Johns, 2019). I continue a life long journey guided by my insights exploring the values of compassion and holistic nursing within curricula (Nathoo et al., 2021). I illustrate reflection as lived sharing stories crafted from everyday life based on my reflective journals.

## Reflection

There many theoretical discussions about the nature of reflection and when writing this paper I take a real-world approach guided by Johns (2017,) description of reflection:

‘Being mindful of self, either within or after experience, as if a mirror in which the practitioner can view and focus self within the context of a particular experience, in order to confront, understand and move towards resolving contradiction between one's vision and actual practice’. (p.3)

Reflection enables individual practitioners to look beyond an experience, informed by literature towards gaining insight into doing things in a better way (Graham & Johns, 2019). For me reflection begins with journal writing, describing and thinking about experiences, exploring emotions and creating a reflective text towards generating a story. The process of gaining insight benefits from engaging with diverse sources (Graham & Johns, 2019). Individual stories generated from experiences illustrate real life creating a narrative, as retrospective meaning making. As Ellis and Bochner (2000) explain:

‘I start with my personal life. I pay attention to my physical feelings, thoughts, and emotions. I try to understand an experience I have lived through. Then I write my experience as a story. By exploring a particular life, I hope to understand a way of life’. (p. 737)

Spear (2002) and Johns (2009) both write of the death of a parent, encouraging me to share stories connecting together a personal and professional life. It behoves me in advancing my commitment to reflection to share my stories as a starting point for dialogue. I therefore seek opportunities when facilitating reflection with undergraduate and postgraduate students. Bringing private grief to a public realm aims to bring to the forefront space to consider individual and professional experiences for nurses who work through the horror of a pandemic. An era where nurses have placed themselves physically and emotionally at risk during Covid-19. It is against this narrative that I suggest the true potential of reflection for me and my work emerges within the chaos described by Jackson et al. (2020) as a ‘tsunami of death’.

Sharing two stories grounded in reflection offers a glimpse of harrowing times of sadness and beauty. Both stories generate a narrative interweaving supporting literature, giving testimony to those who have died and those who have cared for older people during the pandemic. Both stories *Time for Tea* shared with undergraduate and post graduate students and the second story, *Together and Apart,* which is yet to be shared, illuminate a personal view grounded in reflection as starting points to dialogue about our experiences in making sense of caring amidst a pandemic.

Ethical mindfulness pervades the paper and protection of people and place is maintained throughout. I use authentic names, honoring Jim (Dad), Nuala (Mom), Gerry (brother), Vera (sister) and Sarah (daughter, granddaughter). I have permission from family to write these stories as testimony of our lives.

## Story 1: Time for tea

### Setting the Scene

Jim (Dad) is 91 years, and was married to Nuala, my Mom for over 60 years. Now a widower living at home alone, amidst his garden and greenhouse with treasured visits from family. Keeping touch with friends and family via the World Wide Web, Emails fly from near and far. As family we work through grief and treasured memories of Nuala. My brother Gerry lives near, part of Jim's ‘bubble’ with one other household. I live 200 km away and Vera my sister 19,000 km away. In Ireland, older people are asked to cocoon, stay indoors and reduce contact with others as a strategy to reduce the risk of contracting the virus. A safety decree, which may contribute to isolation and loneliness (Jackson et al., 2020). Jim, is stoic. Living a life with minimal physical presence, exacerbating existing problems. Jim's hearing loss becomes ever challenging with growing isolation for all of us, despite trying many technological innovations. We muddle through. For Jim a slow decline in mobility, but holding fast to independence and choices. Orla, [pseudonym] his carer, visits twice weekly and knows Jim's quirks.

#### One Morning

An administrator from the healthcare organization telephones. Jims picks up a muffled message - Orla is on sick leave and a substitute Carer will call.

Soon a carer arrives.

Carer: I am here to give you a shower. Let's go. Your bathroom is upstairs.

Jim: Do we have to do this now?

Carer: I only have a half hour. It is my job.

Afterwards, Jim emails me the following

I am flummoxed. I don't want to lose any help I have.

I hate someone telling me what to do.

I don't want to get the Carer into trouble.

Orla and I have an arrangement.

Usually we start with a cup of tea. Sometimes Orla empties the dishwasher. We discuss the news of the day. We talk of families.

Orla, helps change bed linen while I have my shower (in an adapted bathroom). Orla is nearby. I always feel she never ‘bosses’ me around. I don't have to shower every day.

I know the Carer is only doing her job. I have had relief carers before but this is different’.

Later that evening Jim and I chat over the phone. I mostly listen. It's a struggle. As Gerry Moloney (2020), Church Minister writes ‘For any of us to lose our hearing is a tragedy. It cuts us off from much of life’. I try listening on the phone sending follow up emails. I think of US Senator George Mitchell’s (2004) approach of encouraging a process of listening and listening some more. Covid restrictions limiting me to virtual presence.

An ordinary every day story likely to be repeated anywhere. I feel sad, helpless, watching Jim juggle with choices, stripped to the bone. Where is the progress we have made with caring for older people as individuals? Older people are asked by the Irish Government to stay at home, cocooned without social contacts throughout the pandemic. They are alive but the fundamental question-is whether they are they living? Is keeping people safe, making physical aspects a priority, - the best approach? It is the worst of times amidst Covid-19 crisis. Where is respect and dignity? A rhetoric of forgotten times?

How will people struggling in isolation recover? Jim speaks of ‘the walls can't talk to you’ resonating with the comment of author C.S, Lewis (1961, p. 13) in *A Grief Observed*: ‘I dread the moments when the house is empty’.

What about the little things that matter?

What do we value about people? Where is my life work in promoting human kind practices? I hold on by a thread to an elusive poise. Moving on from feelings of frustration. Trying to stay calm. A grief observed in the midst of Covid-19 world.

It is up close and personal. Torn betwixt and between two places. I am frustrated. Distanced by 200 kilometres. I continue to notice nature, taking daily walks with Dan (husband) appreciating the value of taking time for self. Jim and I continue our nightly telephone calls, talking of rugby, of beloved vines, chrysanthemums, tomato-growing competitions, sharing photographs between Dublin, Melbourne, Wellington and Limerick.

Jim follows virtual journeys weaving across the web. Andrew (grandson) keeps him up to date on his projects.

Celebrating joy about forthcoming first great-grandchild writing in another email

Jim: Best news ever. I look forward to being around next summer.

Nightly telephone calls end with the usual

Jim: Thank you for ringing, get back to your evening with your family

Sometimes I wonder, about my approach. Respecting Jim's choices and rights which have served him well. Avoiding stepping in and taking over. It is never enough, being absent, being virtually present. I hope my listening promotes what an Irish Celtic philosopher, John O’ Donohue (1997) calls ‘a spirit connection of presence’. It is all I can do.

I share my story with students at sessions across different cohorts via virtual learning platforms exploring person centered holistic care. My intention in reading the story aims to give students space to consider nursing values. Inherent in this idea is “‘show not tell’, as thoughts ripple to the surface as a starting point for dialogue with students.

The story seems to reach students, have some impact. Student words ebb and flow across sessions as conversations unfold. The student groups include Year 1 preregistration students in their first academic semester who have no clinical practice; Year 3 students who have had several clinical placements and MSc students who are registered nurses on an advanced practice journey. The story generated dialogue as illustrated in the following snapshots of student responses at sessions and end of semester module feedback.

### Student Responses

Year 1 undergraduate students.

Now I get what person centredness is all about.

Values are important.

Amazing.

That's the sort of nurse I want to be.

It wasn't what she [Orla] did it was how she did it.

I liked the reading it was calm and relaxing I got into the zone.

I like this approach of doing things.

Showing us about reflection will help us learn and prepare for the assignment.

The lecturer spoke of a story about a 90-old man who needs help but did not like the idea of someone helping him in the shower. I thought this story encapsulated what person-centred care is all about.

I thought the story summed up person centred care very well.

It was about mutual respect and allowed the man to shower with dignity.

The relief carer did not show empathy and understanding of the man's preferences and values. The man felt compromised that he would lose help if he did not comply with the demands of the relief carer. The regular carer calmed the situation by listening; acknowledge his situation and offering him a cup of tea.

One key aspect is connecting knowing the little things that matter and not assuming how the person would like to be cared for.

The substitute carer took an opposing approach to the man's care not making a connection with him or asking his showering preferences demanding him to get into the shower.

Leaving the door open slightly that if he needed help she would help him, respecting the man's preference of showering independently and enhanced dignity this is holistic.

I never got the idea of holistic care before, it is more about mind body and spiritual when all together.

This is what I came to nursing for, it's a great way of learning.

I hope to be like the nurse who was kind.

Year 3 students.

It did not take any longer to ‘be with’ Jim

Each module is about different things but here with lecturer's story it comes together

This was a great way to think about older persons and frailty – it's real

MSc postgraduate students

Earlier we had a session on reflection and it was all about cycles this makes more sense

Listening to [MG's] voice… the words got me thinking differently

Lecturer mentioned a key phrase – How would I respond differently? Somehow, while I knew reflection was about learning I didn't have a clue. Now I Get it!

I am surprised about the ordinary simple story as a starting point for reflection

Now see how literature is used in reflection in bring theory and practice together

Our portfolio requires several reflections. I was constantly trying to second-guess what was wanted to get me over the line. Now thinking through my reflections I see that is for me and my practice

In advancing practice, I have been focusing on key skills and capabilities but not paid enough attention to where I am going and being the best I can be. I assume that my ability to really listen is a given. I now need to tease out how I do this more with clients.

I had studied reflection in earlier modules this is a very different slant –more relevant to my work.

### Life Collides

Unraveling my thoughts, I find courage to write about an experience that unfortunately has become a daily reality for individuals, families and health care practitioners during the horrors of Covid-19. I am privileged to share up close personal glimpses. My professional knowledge underscores the value of sharing my personal experience. I write and give voice for Jim's story and for my family, perhaps echoing other people's struggles. I heed the words of the renowned American author Maya Angelou (1969) writing in *I Know Where the Caged Bird Sings* ‘there is no greater agony than bearing an untold story inside you’. Angelou's words give me courage to shift to another focus and draw on my personal knowing. And so begins the crafting of Story 2 extending outwards from *Story 1Time for Tea*. Story 2 is now penned in a public sphere for the first time. It is new. It is raw, and yet to be shared with students. I invite the readers to enter into the spirit of the story. Hopefully, as time goes by I will seek opportunities to generate further dialogue with students and colleagues responses to listening to *Together and apart*.

### Story 2: Together and Apart

#### Early December

Semester finishes, undergraduate and postgraduate assignments graded. Getting ready to share the rituals of Christmas an air of anticipation at what the New Year brings. Looking forward to celebrating Jim's 92^nd^ birthday. Jim is overjoyed at the idea that he would become a great grandfather. All against a continued background of slow decline in mobility and human interaction, controlled by a virus we cannot see.

#### Last Visit

Lockdown restrictions lifted. Sarah [daughter and granddaughter] and I escape to see Jim. We bring tasty morsels, dinners for the freezer, and gifts for everyone. We chat about the everyday, enjoying the rituals of Christmas preparations.

Jim continues as the world lens narrows for all in the midst of pandemic, interested in everything around him embracing technology. Yet, hearing loss interfering with family connections.

As we leave

*MG [author]* Take care we will be back

*Sarah* Love you to the moon and back [their catch phrase].

#### Mid December

A call, Jim has fallen, knee Injury flares up reducing mobility. Now in hospital. Nurses help Jim log on to the hospital internet. We receive emails saying care is excellent.

Every 48 h negative Covid-19 tests.

A positive test. Hospital acquired Covid-19.

Jim a growing statistic. Emails back and forth.

We try to show our love, no vision, no hearing, no presence, no touch. My earlier readings of O’ Donohue (1997) haunt me about touch as bringing presence home in expressing compassion.

#### Christmas Eve

Jim's last email

‘The nurses are fantastic enjoy yourself don't mind the old codger, will keep fighting this.

Love you heaps’.

Gerry, designated contact with hospital teams circulates daily telephone calls along family networks.

In the meantime, Covid-19 numbers ever rising, mounting pressures. Second lockdown coming. Hospital ward phones ring out. Later, we hear some people make over 80 telephone calls unanswered. It must be a nightmare for staff working Christmas shifts. Rather than frustration with systems, I know the procedure, feeling privileged to receiving any news. Some nurses acknowledge Jim as an individual person.

No visitors allowed. Hugelius et al. (2021) in a integrative review conclude that visiting restrictions, may have a negative impact on patients, families, and health care services beyond the pandemic and nurses need to adapt care to compensate for such effects.

Gerry, Vera and I connect via *Google Meets* expressing our fears and concerns about Jim being alone, across time zones together and 19,000 Kilometres apart.

We try to get messages to him through the hospital communication office, closed ‘til after Christmas.

Jim has ‘special’ nurses in a room of his own.

The best we can hope for, if we are privileged, is a visit.

My life's work ebbs and flows. Not being able to visit, not to let him know we care. Person centredness eludes me. Taking time to pause, noticing my feelings, no anger, simply, acknowledging the unimagined horror of the alien world of a Covid-19 ward.

#### Crisis Looms

Day 8, following diagnosis of Covid-19 the focus on scientific management disease trajectory, oxygen saturation stats falling, antibiotics and steroids. Are staff retreating in a protective shell of medical jargon immune to person centredness? Do they know we want to hear about Jim? Kitson et al. (2021) encourage nurses to be present for patients in a spiritual sense, given the nurse may be the only other person in contact, acknowledging the difficulties in providing holistic care while working in such dire situations.

Situation worsens. Our fears are realized, Continuous Positive Airway Pressure management (CPAP) begins.

The discomfort of the mask intensifies.

Jim is getting tired.

We know Jim is organized and understand that hospital teams have had conversations with him about his choices.

#### The Last day

December 30^th^ Jim's birthday.

The call comes while walking with Dan, crossing a bridge over the Shannon River, usually a view that cheers. I watch the gray churning depths of the fast flowing water, sensing that I am passing into a vortex. Later, dusk falling, darkness creeping into our bones, we take time to visit a favorite church, open for private prayer during Covid-19. Lighting a candle at a treasured icon, known to Jim.

Jim is suffering, struggling with CPAP, he agrees no further interventions.

Waiting for the inevitable. A surreal experience. Albuquerque et al. (2021) argue that Covid-19 has altered the landscape of grief adding to suffering. Bereaved individuals may feel guilty, being absent at the time of death or unable to provide comfort. Feelings may intensify in the context of the pandemic. I strive to be practical and appreciate the tension around safety and my wish to be present.

Gerry is allowed visit Jim. Dressed in protective gear, facilitates *WhatsApp* calls from everyone, lighting the screen connecting our worlds in Ireland and beyond. New Zealand and Australia. Jim sees names.

The sentiment - love you [Jim] to the moon and back.

*Gerry* Jim wants to go asleep, no more face mask

Nurses are managing Jim’ discomfort, he is at peace.

Vera: Jim is in control. That is how he would want it.

In typical Irish fashion, nurses bring tea, popping in and out.

Jim shows Gerry lists. Everything ready, affairs in order. Meticulous to the end. An approach similar to descriptions by Spear (2002). Jim worries that Gerry might get Covid-19 and pleads with Gerry to go home.

Gerry: Jim's waiting for the *Last Rites,* blessing and Catholic rituals for the dying, administered by a Chaplin.

We wait. Beside me a photo of Jim and a candle lighting.

We watch, keeping a vigil

Vera: Gerry is the sentinel for us all

I keep knitting, a baby cardigan as the cycle of life continues.

The macabre and the mundane. Many dropped stitches along the way.

#### Into the Night

Gerry: Jim wants to sleep. He is calmer, pain relief from Morpheus helps.

#### New Year's Eve

Jim lives ‘til his birthday is over. It is past his bedtime and he does not keep us up all night.

Jim was one of 11 who died on that day in Ireland

As the U.S. National Institute of Aging (2020) summarizes people who are dying need care across four areas—physical comfort, mental and emotional needs, spiritual issues, and practical tasks.

On the last evening in the presence of Gerry, Jim died with limited presence as Kearney (2021) notes:

The last thing we do when dying is to reach for another hand, something that the pandemic has made impossible for so many. We need to get back in touch with real people and things (p.4)

#### Funeral Rites

In a winter of growing despair, we prepare for the rituals surrounding death robbed of the traditional comfort of Irish customs. Another casualty of Covid-19 (Albuquerque et al., 2021). Nevertheless, we end the celebration of Jim's life, following strict guidelines, only 10 people present. We followed every guideline, we owed it to Jim. We kept him safe and then life happened and a hospital acquired virus attacked. Standing two metres apart, not allowed to hug or exchange a traditional Irish hand shake. Close family unable to travel as borders closed. Many friends and extended family missing. Limited opportunities to speak of Jim, show compassion or support in person. Further possibilities of a more complex response to bereavement (Albuquerque et al., 2021). We share video links of services. Finding solace in the final music choice *Ode to Joy* composed by Beethoven when profoundly deaf. Johns et al. (2020) argue that for families Covid-19 related deaths, are lonely and dehumanized processes, with a possibility of disenfranchised grief whereby bereavement and the associated social mourning rituals are compromised adding further complexity to the grieving process.

My narrative, while individual to me, is similar to many other people's experiences. For me, the nursing team tried to ease Jim's suffering. Whether all of us were present now seems immaterial to me. What is important for me, is that I believe that Jim knew we were with him in spirit. That is what I hold on to, where my personal and professional world of the science and art of nursing cohere.

### Insights

So far, I illustrate reflective journaling in generating my stories. Several insights emerge as I unravel my understanding of grief and loss. I draw on diverse forms of knowing spanning empirics and esthetics grounded in my personal knowing, choosing to focus on four elements within the narrative. I begin with the health impact of Covid-19 on nurses’ endeavors to provide holistic care, identified through traditional research and media communication. Then I turn to comment on courage in telling stories though virtual platforms and consider self-care and reflective writing as restorative practice.

Globally science tells the facts. Covid-19 figures highlight that in one study 41% of patients presumed to have transmission in hospital (Wang et al., 2020). Late January, 2021, numbers in Ireland escalate with 91,779 cases confirmed (Department of Health Government of Ireland, 2020). Global reports reveal stark realties. From China a study by Nie et al. (2020) conclude that front-line nurses, working at COVID-19 units suffered from increased psychological distress. From Italy, Catania et al. (2021) report on nurses caring for critically ill or dying colleagues whereby nurses were living apart from their families to protect them from infection. From Iran, Kakemam et al. (2021) identify increased burnout recommending nurses requires access to psychosocial support, including web-based services, psychological aid and self-care techniques. Findings are submerged in epidemiology statistics and safety in reducing impact of virus spread. Media talk concentrates on pressures on Intensive Care Units, less attention to the support for people dying and those caring for them. In the United Kingdom the situation is escalating as Lewis Goodall, BBC policy analyst, tweets:

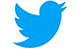

**Lewis Goodall** @lewis_goodall Dec 28, 2020

Been speaking to several London frontline doctors working in major hospitals. Picture isn't great. One: “it's a bit like a warzone. I’d say not far off where we were at in first wave in terms of capacity. But staff are completely demoralised and exhausted this time round.”

From the United States. Dr Esther Choo shared a still image from a news cast quoting 1,761,749 global deaths and tweets:

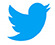

Esther Choo MD MPH, @choo_ek,· Dec 28, 2020

We are drowning in patients across the US. Right now 1 in 1500 Americans over the age of 25 is hospitalised with Covid. We aren't designed for that and we can't take much more.

These typical media comments are supported by The Kings Fund (2020) report that the impact of the pandemic on nursing workforces will be felt for a long time. Galvin et al. (2020) report that student experiences of the high numbers of patient deaths without adequate preparation leads to increased risk of mental health problems. Nurses need effective support, including reflection to flourish in caring for those who are dying and helping with grief requires further consideration for facilitating learning.

Creating meaningful learning spaces has been troublesome within the loss of face to face student interaction required to maintain safe environments during the pandemic. I gradually become more confident in facilitating virtual learning platforms and engage with learning technologists. Culp-Roche et al., (2021) report that successful virtual teaching educators require support and confidence in creating learning material that students can understand and apply to practice. There have been glitches and sometimes poor internet connection. Early thoughts concentrated on simple virtual platform management of sequence and content. I persist, keeping true to my values. I walk the walk as an educator showing that reflective writing is embedded in my work rather than simply espouse theories of reflection. Taking courage, I lean towards stories as simple performance, giving students space and time to listen and derive meaning from stories of compassion (Turkel et al., 2018). Student responses are positive. There is need to give further consideration to seeking and receiving feedback from students. Snapshots presented here affirm my life long endeavor in seeking ways to connect with students in promoting holistic nursing illustrating the potential of reflection.

An impassioned call to practice self-kindness as fundamental to caring for another is illustrated by the international scholar Jean Watson (2020), commenting: ‘At this time of shock and world change we are called to surrender — returning to wisdom, to spiritual, redefining our reality, what truly matters, by the focus on that which is lasting from within’. 

I have learned to take time to enjoy walking along seashores, rivers, becoming aware of surrounding nature, watching weather patterns, changing seasons taking moments to be intentionally situated in the here and now as suggested by Nilsson (2021). Approaches that I see as inherent in Celtic spirituality, akin to mindfulness as the first cue in-Johns model of reflection- *Bring the mind home,* aiming to develop poise and nurture emotional intelligence (Johns, 2017, p. 21). Poise is an attribute contributing to understanding, managing emotions in positive ways and minimizing response to stress, overcoming challenges and developing resilience. Attributes inherent in the concept of emotional intelligence (Goleman, 1998). I become more aware of the influence of emotion as a stressor that may interfere with being empathetic. I leave behind the horror, frustration and anger, rather than letting them overwhelm me, instead recalling beautiful memories. I pause, take a moment, acknowledging the emotions of loss and grief. I know that over time there will more tidal waves of grief ebbing and flowing. I focus on the here and now taking a deep breath, noticing nature, becoming more mindful congruent with Johns approaches (Graham and Johns, 2019). Barnett et al. (2021) report on positive findings from an innovative project in addressing personal care needs of nurses whereby a shift was demonstrated in moving from fear and fatigue and stress towards more mindful caring presence. Barnett et al. (2021) note the significance of nurses risking their lives to provide compassion to patients, within the pandemic, while dealing with their own stress, anxiety, compassion fatigue, and burnout. Mc Callum et al. (2021) argue that nurses’ stress intensifies in a struggle to deal with professional grief and with the loss of being able to provide the very best of holistic nursing care.

Writing this paper began with a sense of uncertainty. The US educationalist bell hooks (2015), too writes of uneasiness, of reluctance when developing work. My thoughts wander back and forward about the appropriateness of sharing Jim's story. Jim liked my earlier reflective writing. My parents were together over 60 years. I know that I am privileged having witnessed lives of parents living so well for so long. I try to write with honesty, with family support. It has been cathartic, sometimes sad, and sometimes joyful. I share Jim's story to help give voice to those who have no voice and to advocate for people who are dying (Spear, 2002). We imagine that our stories are so out there, so unique that nobody else will be able to relate to them. Perhaps the opposite is true. As Schwind and Manankil-Rankin (2020) suggest narrative processes open a relational space for a co constructed space with readers.

My narrative aims to disturb our thinking, drawing attention to a hidden nursing world amidst a pandemic. Previously shared stories include a celebration of nursing witnessed when my Mom died (Graham, 2018), at odds with Johns’ experience of the *Death of my mothe*r (Johns, 2009). In the midst of a season of darkness, I keep writing. I find comfort in writing. It comes quickly. I realize I miss expressing my thoughts and emotions about experiences in my reflective journal. No entries in my journal for early winter. Now returning to the comfort of writing and creating these stories, celebrating beauty juxtaposed with horror amidst a pandemic. Such writing goes beyond technical knowledge not traditionally described in textbooks (Schön, 1983). For me reflective writing has potential in fostering self-care as a foundation for revisioning caring for others in harrowing times. My stories begin a restorative and healing process for me, connecting together inner dimensions of self in harmony with an outer self in understanding my role as a person as a holistic nurse educator (Rew, 2005). The wounds of Covid- 19 run deep and as the emphasis shifts to vaccination and new variants, raising more and more questions. Are we immune to suffering? How do we reconnect with people and in particular older people lost in a morass of isolation? How do we heal? How do we encourage nurses to flourish? There are many possibilities reported in the *Journal of Holistic Nursing*. Insights are presented as I understand my experiences of grief and loss, are partial with many other layers to be unraveled. No doubt there will be other interpretations as time goes by.

## Reflexivity

Reflexivity recognizes that we are always present in our writing and that separating ourselves from personal knowledge and experience is difficult (Faulkner et al., 2016). Reflexivity is looking back, connecting experiences, and making sense of emerging insights, embedded in the context of the unfolding work full of learning opportunities. As American poet T.S. Eliot (1963) in *Little Gidding* eloquently expresses

‘We shall not cease from exploration and the end of all our exploring will be to arrive where we started and know the place for the first time.’(p. 222)

Uncertainty about the integrity of my writing ripple forth. It shifts from indulgence looking inwards without the true essence of reflection, towards looking outwards, enriching my life and responding differently informed by insights. Illustrating coherence in reflective writing is challenging and may not capture the entirety, given the nature of the struggles therein. Establishing the authenticity of narratives is troubling as insights are tentative and partial, grounded in subjectivity around whether I am true to my reality. Ellis and Bochner (2004) suggest that stories cross boundaries between social science, literature and the arts helping make the personal political. Such ideas have resonance for me in enhancing my role as a holistic nurse educator. Bocher and Ellis (2004) ask the questions. Does the narrative seem real and true? Does the paper tell a story? Conclusions, surrounding the coherence and authenticity of the work rest with the reader.

## Afterword

I share a glimpse of a narrative of a celebration of Jim's life, of decline, grief and loss as a daughter and as a nurse educator. Perhaps my stories resonate with readers’ lives as students, nurses, as an individual and a member of a family, illustrating caring science. We need to share and listen to more stories. Together stories become a narrative that honors the people of the past and present, beyond the horror of the pandemic. Covid-19 statistics confirm 4,235,559 deaths (WHO, 04 August 2021). Stark statistics represent individuals, families, colleagues and communities with an ever widening impact on global health. My father was one of eleven who died that day. An individual hidden in a daily statistic. As our world focus shifts to new variant waves and global vaccination we must keep exploring learning strategies in supporting nurses towards recovery from the trauma of caring for people in the midst of a pandemic.

The paper aims to be a starting point for global dialogue about holistic nursing. It invites the reader to take time to consider the potential and implications of reflective writing and storytelling as a strategy contributing towards educating and helping nurses to flourish. Thinking about experiences, writing, sharing memories, exploring literature towards enhancing practice is a lifelong journey. Take time for self-care as a starting point for a reflective journey. Reflection as thus understood offers an approach to reconnect the art and science of nursing, showing promise for nurse educators in weaving the mystery and artistry of praxis enabling us to make sense of a world of chaos while together and apart.
